# Quorum sensing influences growth and photosynthetic membrane production in high-cell-density cultivations of *Rhodospirillum rubrum*

**DOI:** 10.1186/1471-2180-13-189

**Published:** 2013-08-08

**Authors:** Lisa Carius, Anke B Carius, Matthew McIntosh, Hartmut Grammel

**Affiliations:** 1 Max Planck Institute for Dynamics of Complex Technical Systems, Sandtorstr. 1, 39106, Magdeburg, Germany; 2 Loewe Center for Synthetic Microbiology, Philipps-University Marburg, Hans-Meerwein-Str. 6, 35043, Marburg, Germany; 3 Biberach Universtiy of Applied Science, Karlsstr 11, 88400, Biberach, Germany

**Keywords:** *Rhodospirillum rubrum*, Quorum sensing, Acylhomoserine lactones, High cell density cultivation, *Lux* genes

## Abstract

**Background:**

The facultative anoxygenic photosynthetic bacterium *Rhodospirillum rubrum* exhibits versatile metabolic activity allowing the adaptation to rapidly changing growth conditions in its natural habitat, the microaerobic and anoxic zones of stagnant waters. The microaerobic growth mode is of special interest as it allows the high-level expression of photosynthetic membranes when grown on succinate and fructose in the dark, which could significantly simplify the industrial production of compounds associated with PM formation. However, recently we showed that PM synthesis is no longer inducible when *R. rubrum* cultures are grown to high cell densities under aerobic conditions. In addition a reduction of the growth rate and the continued accumulation of precursor molecules for bacteriochlorophyll synthesis were observed under high cell densities conditions.

**Results:**

In the present work, we demonstrate that the cell density-dependent effects are reversible if the culture supernatant is replaced by fresh medium. We identified six *N*-acylhomoserine lactones and show that four of them are produced in varying amounts according to the growth phase and the applied growth conditions. Further, we demonstrate that *N*-acylhomoserine lactones and tetrapyrrole compounds released into the growth medium affect the growth rate and PM expression in high cell density cultures.

**Conclusions:**

In summary, we provide evidence that *R. rubrum* possesses a Lux-type quorum sensing system which influences the biosynthesis of PM and the growth rate and is thus likely to be involved in the phenotypes of high cell density cultures and the rapid adaptation to changing environmental conditions.

## Background

Quorum sensing has become an important aspect of microbiological research in the last 30 years. An *N*-acetylated homoserine lactone (AHL) based quorum sensing system was first discovered in *Vibrio fischeri*[1]. *V. fischeri* can either live freely in the ocean or undergo commensalistic relationships with deep sea fish, where they populate light organs at high population densities. Only at appropriate population densities is luminescence production triggered by the Lux quorum sensor system. It consists of an AHL synthase, LuxI, which is responsible for the formation of the autoinducer 3-oxo-C6-HSL. This autoinducer binds to the response regulator, LuxR, which then binds to a specific DNA motif called the Lux box. The AHL-LuxR-DNA binding results in the regulation of expression of the *lux* genes responsible for luminescence. Additionally, the AHL-LuxR complex also enhances the expression of *luxI*, leading to the increased rate of AHL production. AHLs are typically produced at a constitutive rate at population densities below the ‘quorate’. In this way, the AHL concentration is kept in proportion to the population density. When the AHL concentration reaches a threshold, LuxR becomes active and increases the expression of *luxI* and thus AHL production. At that point, quorum sensing regulation begins [2,3].

*Rhodospirillum rubrum* is an anoxygenic photosynthetic bacterium which has served as a model organism for cellular redox studies during the last decades e.g. [4-7]. These bacteria are of special interest for biotechnological applications, as they are the only known species of its kind which produces maximum amounts of intracytoplasmic photosynthetic membranes (PM) under microaerobic conditions in darkness when grown with succinate and fructose (M2SF) as carbon sources [4,5]. Using this light-independent cultivation system for the industrial production of PM could highly simplify the biotechnological synthesis of a number of interesting compounds, which associates the formation of PM, such as pigments, vitamins and coenzymes [6,7]. In this context Sasikala *et al.,* who reviewed the ability of using anoxygenic photosynthetic bacteria for industrial application, pointed out that almost all of these studies were conducted under phototrophic conditions using light as energy source. However, since high productivities of biotechnological large scale applications depends on attaining high cell density conditions the light input becomes a strong yield limiting factor [8]. Clearly, using *R. rubrum* as potential producer organism of PM-related compounds could bypass these problems. The recent demonstration of lycopene production in *R. rubrum*[9] or the development of an expression system for heterologous expression of membrane proteins [10] are further examples of the attractivity of this bacterium as a producer in biotechnology given that large scale cultivation at high cell densities can be achieved.

Recently, indications of quorum sensing related behavior appeared in fed-batch cultivations with *R. rubrum*[11]. Zeiger and Grammel found that at high cell densities (HCD), PM synthesis was no longer inducible by reducing the oxygen supply of the cells. Limiting oxygen conditions (microaerobic or anaerobic) are generally the major environmental factor for inducing PM biosynthesis.

There has been some published work on quorum sensing systems in photosynthetic bacteria. In *Rhodobacter sphaeroides* 7,8-cis-N-(tetradecenoyl)homoserine lactone was identified previously as an AHL signaling molecule, involved in colony morphology and cell aggregation [12]. Interestingly, a new class of AHL appeared in *Rhodopseudomonas palustris* where p-coumaroyl-homserinelactone was combinatorially synthesized with bacterial homeserinelactone as one building-block and plant-derived p-coumaric acid taken from the environment as the other [13]. Furthermore, AHLs have also been detected in cultures of several aerobic anoxygenic phototrophs [14]. Although these examples suggest that AHL production in alpha-proteobacteria is the rule rather than the exception, there is up to now, no report of an AHL molecule present in *R. rubrum*.

In this study, we present evidence for a Lux type quorum sensing system in *R. rubrum* responsible for the production of at least four quantifiable AHL species that influence growth rate and PM formation. This organism contains versatile metabolic activity and therefore exhibits variant growth behavior dependent upon the availability of carbon source, oxygen tension and light intensity. We investigated quorum sensing in the aerobic, microaerobic and anaerobic phototrophic growth modes, each of which results in the production of differing amounts of PM.

## Methods

### Bacterial organism and growth conditions of batch cultivation

*R. rubrum* strain *ATCC 11170* was cultured under aerobic, microaerobic and anaerobic phototrophic conditions on M2SF medium at 30°C. The M2SF medium was based upon the minimal M medium introduced by Sistrom [15] and contains 40 mmol L^-1^ succinate and 16.6 mmol L^-1^ fructose as carbon sources [4]. Bacteria were grown in aerobic and microaerobic conditions in shaker flasks with 3 baffles on a Certomat BS1 rotary 124 shaker (Sartorius, Goettingen, Germany) at 100 rpm in the dark. To ensure adequate air supply under aerobic conditions, only 10% of the flask volume was occupied with culture medium, whereas oxygen-limited (microaerobic) conditions were obtained by occupying 50% of the flask volume with liquid medium. Anaerobic photosynthetic cultures were grown in filled Pyrex flasks illuminated with tungsten light bulbs with approximately 15 microeinsteins m^-2^ s^-1^ and stirred with a magnetic stirrer at 260 rpm as described previously [5]. All cultivations were started with an initial optical density (OD) of 0.1.

### Bioreactor cultivation

To obtain controlled process conditions, bioreactor cultures were grown under aerobic and microaerobic conditions in the dark in stainless steel bioreactors (Biostat C; B. Braun Biotech, Melsungen, Germany) with a 5-liter working volume. Process parameters were controlled with a Simatic PCS7 automation system (PSC7-V6.0, Siemens, Munich, Germany). The temperature was kept constant at 30°C, and the agitation rate was 250 rpm. The pH, measured with a glass electrode (405-DPAS-SC-K8S/325, Mettler-Toledo, Langenfeld, Germany), was kept at pH 6.8 using 1 M KOH or 0.66 M H_3_PO4. Under aerobic conditions dissolved oxygen was monitored using a fiber optic oxygen sensor, with a measurement range of 0 - 20% partial oxygen pressure (pO_2_) (Fibox 3-Trace, PreSens, Regensburg, Germany) and controlled at 2% pO_2_. To monitor and control microaerobic conditions, the culture redox potential (CRP) was measured by an *in situ* oxidation-reduction probe (Pt4805-DPAS-SC-K8S, Mettler-Toledo, Urdorf, Switzerland) connected to a voltage transmitter (pH-2100 transmitter, Mettler-Toledo, Urdorf, Switzerland). For a detailed description of the CRP-dependent control strategy, cf. [16]. The oxygen supply was adjusted by varying the inlet gas composition (in-house construction based on a gas-mix station module of Bronkhorst Maettig, Kamen, Germany) with N_2_ and air as inputs. The flow rate was kept constant at 1 L min^-1^ (0.272 vvm). To obtain high cell densities (HCD), cells were cultivated in a Fed-Batch operation mode. The feeding strategy was accomplished by open loop control using an exponential feeding profile [17] which was slightly modified from that as described previously [11].

### Growth experiments with Fed-batch aliquots

A 50 mL aliquot of culture broth was taken from Fed-Batch cultivations at different ODs under sterile conditions. The aliquot was centrifuged at 5000 × *g* for 10 min at room temperature to separate the cells from the culture supernatant. Cells were then washed in 0.98% (w/v) sodium chloride under sterile conditions, resuspended in fresh M2SF medium and then further cultivated under microaerobic conditions. The culture supernatant was first filtered (Minispike Acrodisc® Syringe Filter, 0.2 μm, GHP, Pall Life Sciences, New York, USA) to remove insoluble material, then inoculated with cells obtained from a standard preculture and the culture was further cultivated under microaerobic conditions.

### Spectroscopic methods

OD (660 nm) and PM levels (880 nm) were measured using a 1 cm path length cuvette and a UV/Vis spectrophotometer (V-560, Jasco, Tokyo, Japan). The PM level was estimated using the A880/A660 ratio. An A880/A660 ratio of approximately 1.2 is characteristic of maximal PM levels, obtained in anaerobic phototrophic cells grown at low levels of light intensity. An A880/A660 ratio of approximately 0.54 is indicative of a lack of PM formation, and occurs in aerobic cultivation conditions [4]. ΔPM refers to the amount of PM produced during a specific growth period. Culture supernatants were analyzed for levels of bacteriochlorophyll *a* precursors by fluorescence spectroscopy using a Varian fluorescence spectrophotometer of the type Cary Eclipse (Cary Eclipse, Varian, Palo Alto, CA). Tetrapyrolle compounds produced in growth cultures were identified as described previously [11]. For quantification of both compounds, the emission spectra of culture supernatants were evaluated at their maximum emission (FI_max_). Protoporphyrin-IX (PPIX) showed a FI_max_ at 614 nm when excited at 390 nm, whereas magnesium-protoporphyrine-IX-monomethylesther (Mg-PPIX-mme) showed a FI_max_ at 595 nm when excited at 420 nm.

### Purification and quantification of AHL extracts

Culture supernatants were extracted with dichloromethane in a ratio of 7:3 (v/v). After evaporation of the solvent, the dried AHL residue was resuspended in 100% (v/v) acetonitrile (ACN) at 1/100 of the origin volume. In preparation for analytical high performance liquid chromatography (HPLC) analysis, the samples were filtered (0.2 μm, GHP, Minispike Acrodisc® Syringe Filters, Pall Life Sciences, New York, USA) to remove particulate matter. The samples were processed on a HPLC from Agilent (1100 series, Agilent, Waldbronn, Germany) consisting of quaternary pump, autosampler, DAD-detector and the matching LC/MSD detector or a 1200 series sample collector. The LC/MSD (1100 series, Agilent, Waldbronn, Germany) was used with either an APCI-ion source or ESI. The Inertsil ODS-3 column was 4.6 x 250 mm, with a 5 μ particle size (Inertsil 100A ODS-3, VDS Optilab, Berlin, Germany). The eluent gradient was from ACN:H_2_O; (10:90; v/v) to ACN:H_2_O (90:10; v/v) over 15 min. For restoring the original concentrations between samples, a 5 min flow interval, followed by 3 additional minutes for equilibration was used. For sensitive analysis, the flow rate was 1 mL min^-1^. For semi-preparative applications involving a larger column (10 x 250 mm), the flow rate was adjusted to 3 mL min^-1^.

### Screen for AHL bioactivity

Autoinducer bioassays [18] were performed employing *A. tumefaciens* NTL4 (pZLR4) as indicator strain. The overlay culture was prepared as described previously [19]. An appropriate amount of AHL extracts was spotted on glass microfibre filters (90 mm Ø, Cat No 1822–090, Whatman, GE Healthcare UK limited, Little Chalfont, UK) which were then placed into a Petri dish. Filters were overlaid with top agar containing the indicator strain. Plates were incubated for 8 hours at 30°C.

### Thin layer chromatography (TLC) of AHLs

Respective amounts of samples were spotted on C18 reversed-phase TLC-Plates (Merck, Darmstadt, Germany) and dried with a cold air fan. The chromatography was processed in a chamber filled up to 1 cm with a mixture of methanol and water (60:40 v/v). After the solvent was 1 cm from the top of the plate, the plate was taken out and the solvent was allowed to evaporate. The dry plates were placed in petri-dishes and covered with top agar containing the indicator strain *A. tumefaciens* NTL4 as described above.

### Analysis of mRNA levels

Cell samples were directly treated with RNA protect (RNA protect Bacteria Reagent, Quiagen, Hilden, Germany). RNA was isolated according to the NucleoSpin RNA II protocol from Macherey-Nagel (5.3 Support protocol NucleoSpin RNA II, Machery-Nagel, Düren, Germany) and stored at −80°C. For cDNA synthesis of the target genes, 500 ng total RNA of each sample was transcribed applying the reverse primers (for primer sequences see Additional file 1: Table S1). Each primer contained a 20 bp match to the target gene. The reverse transcriptase step was performed in triplicates (RevertAid^TM^ H Minus M-MuLV Reverse Transcriptase, Thermo Fisher Scientific, Vilnius, Lithuania). RNA was tested for DNA contamination prior to cDNA synthesis by using the total RNA isolate as template for the real time PCR. Real time PCR of the pooled cDNA preparations was conducted using the SYBR® Green PCR master mix from Life Technologies (SYBR Green 1 Dye, AmpliTaq Gold® DNA Polymerase, Life Technologies, Carlsbad, USA). The gene encoding 16S rRNA (Rru_AR0004) with the primer pair Fwd: AGGTGACACTATAGAATATACGGGAGGCAGCAGTGGGG, Rev: GTACGACTCACTATA GGGATCACTCACGCGGCATGGCTG) was used as housekeeping gene which proved to be constant at all tested cultivation conditions. All real time PCR data were obtained from 5 biological replicas (cultivations) under aerobic, microaerobic and phototrophic conditions, respectively. From each cultivation, 3 × 250 ng total RNA were amplified using specific primers. The three resulting cDNA molecules/sample were pooled and 3x determined by real time PCR. mRNA amounts were estimated using the experimentally determined efficiencies according to Pfaffl et *al.*[20].

### Cluster analysis

Cluster analysis was performed using the open source software PermutMatrix version 1.9.3 [21]. For this purpose data sets were first processed by Z normalization. Dissimilarity between the data sets was calculated applying the Pearson Distance, which calculates the correlation of a linear relationship of two variables. Once the calculated residual errors were taken into account, the data set was then hierarchically clustered applying the Wards minimum variance criterion [22]. According to this method pairs were merged in clusters such that the total error within a group was minimized.

## Results

### High cell density repression of PM biosynthesis - quorum related?

**Figure 1:**
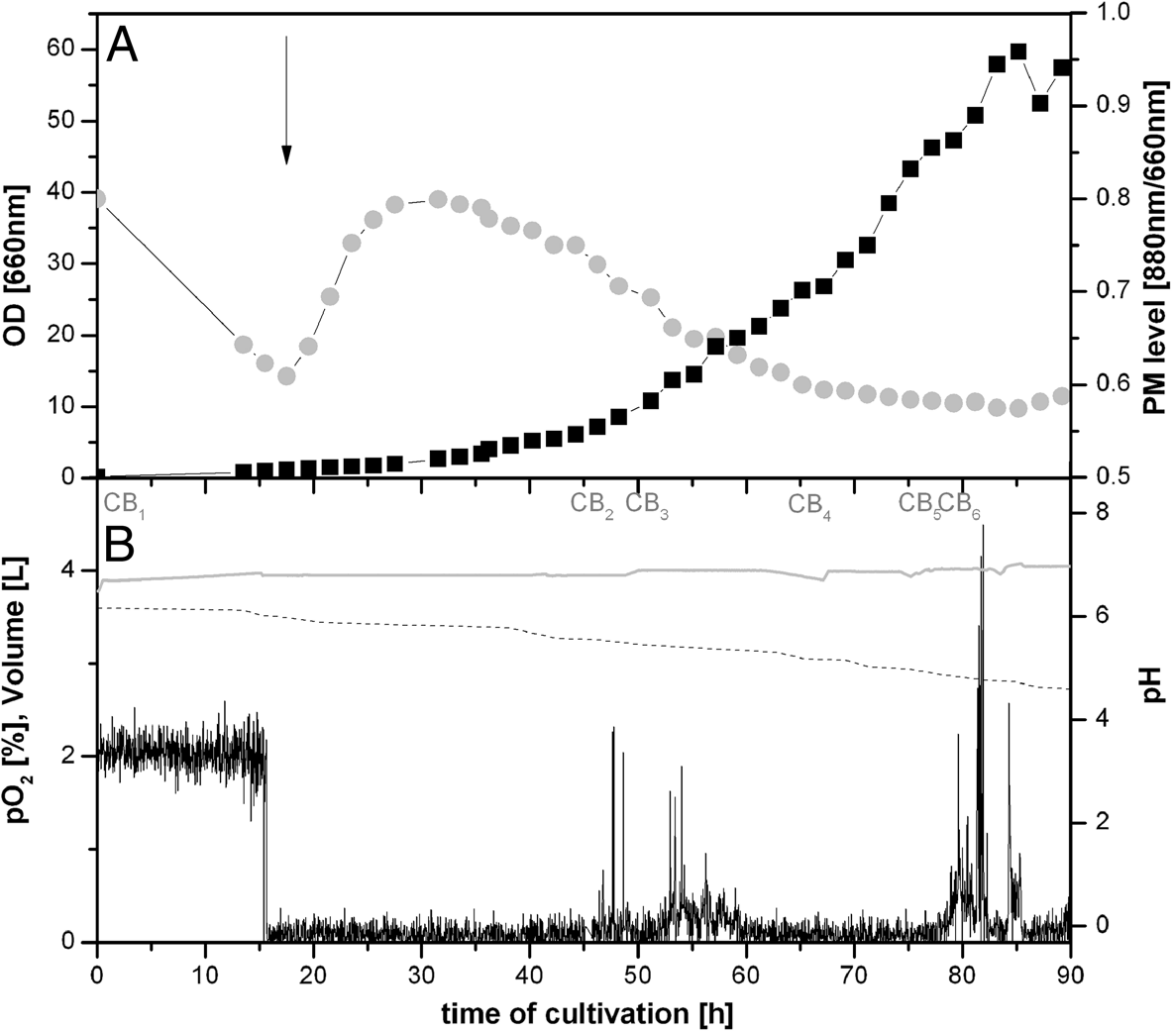
**Microaerobic Fed-Batch HCD cultivation of *R. rubrum*. A**: OD (660 nm, ■) and PM levels (880/660 nm, gray circle symbol). Time points where samples were taken for further cultivation experiments are indicated as culture broth (CB_1-6_). **B**: pH (gray line), partial oxygen tension pO_2_ (—) and total culture volume (− −). The shift in oxygen availability was induced at 15 hours, indicated by the arrow.

Recently, we reported that the production of PM was halted in HCD cultivations of *R. rubrum*. When *R. rubrum* cells were initially grown aerobically to an OD >100 and then shifted to conditions optimal for PM synthesis, i.e., oxygen limitation, no PM formation was observed [11]. In the present study, we observed that when the cells were shifted at a lower population density to microaerobic conditions PM synthesis stagnated at an OD <10, continuously decreased parallel to the culture growth rate and was completely inhibited at an OD ~30 (Figure 1A), even though oxygen remained the sole growth limiting factor (Figure 1B). In this experiment, oxygen levels were maintained at microaerobic levels by process control of the culture redox potential (CRP) [16]. The carbon sources, succinate and fructose, were supplied in excess throughout the cultivation by Fed-Batch operation of the bioreactor. Interestingly, complete inhibition of PM synthesis after ~60 hours coincided with the accumulation of protoporphyrin IX (PPIX) and Mg-protoporphyrin IX- monomethylester (Mg-PPIX-mme) in the supernatant. This effect occurred in all microaerobic HCD cultures independently of whether CRP or partial oxygen pressure (pO_2_) were employed as controlled process variables. The observed impairment of PM expression at high cell densities could either result from soluble inhibitory factors accumulating in the culture broth or from genetic or regulatory alterations. One or more mutations in genes responsible for PM biosynthesis is one such possibility which could provide a selective growth advantage in chemotrophic Fed-Batch cultivations.

**Figure 2:**
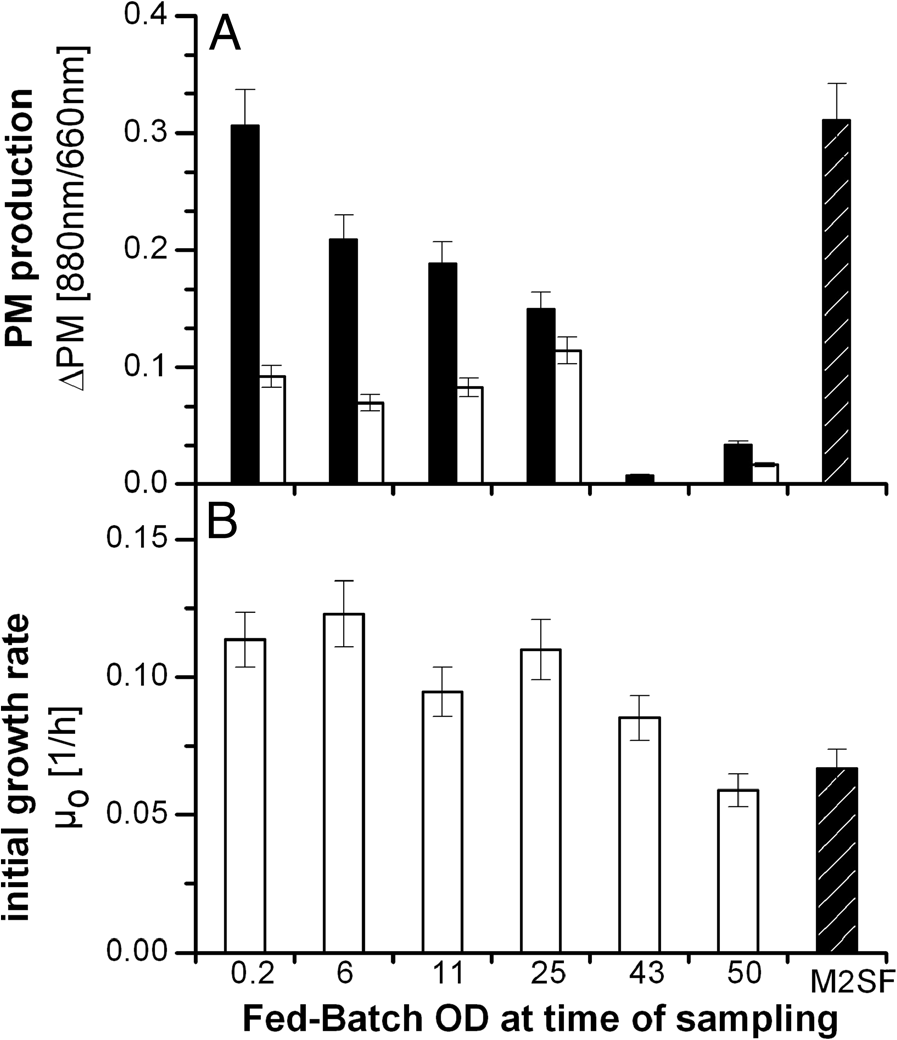
**Effect of culture supernatants, obtained at various optical densities, on photosynthetic membrane production (A) and cell growth (B) of *R. rubrum. *A**: PM production during microaerobic cultivation using sterile filtered culture supernatants and cells harvested from an aerobic Fed-Batch cultivation. Black bars represent production in cells harvested from the Fed-Batch cultivation, washed and resuspended in fresh medium. White bars indicate cells harvested from an aerobic pre-culture, washed and resuspended in supernatant from the same Fed-Batch cultivation. **B**: Initial growth rate under microaerobic conditions after cells were inoculated into filtered culture supernatant harvested from the same aerobic Fed-Batch cultivation. As a control for both **A** and **B**, cells harvested from an aerobically grown preculture were washed and resuspended in fresh medium (striped bars). Rates were calculated from data during the growth phase of the cultivation. The shown data represents the mean of three measurements. Error bars were calculated by error propagation with accumulated deviations of three equivalent experiments. (Cells and culture supernatants from three Fed-Batch cultivations were treated as described above).

A series of experiments was therefore conducted to examine both possibilities. Cells were taken from Fed-Batch cultivations at varying OD levels, washed in 0.98% (w/v) sodium chloride under sterile conditions and resuspended in fresh cultivation medium (M2SF medium). Simultaneously, the filtrated supernatants of the same Fed-Batch samples were inoculated with new *R. rubrum* cells from an aerobic preculture. In a control culture, PM expression was induced when microaerobic conditions were reached due to oxygen consumption by cell growth at OD = 1, as expected. All cultures were grown under microaerobic conditions in shake flasks until their stationary phases. The results presented in Figure 2A show that in the resuspended Fed-Batch cells, a sharp decline of PM production occurred with increasing cell densities of the harvested cells. Note that the original Fed-Batch OD at the time point of sampling was the initial OD of the resuspended cultures (black bars in Figure 2A). The amount of PM production in cells harvested at OD = 0.2 were comparable to the control culture whereas only negligible amounts were observed in cells harvested at ODs above 40. An inhibitory effect was also observed when Fed-Batch culture supernatants were applied as cultivation medium for fresh cells (white bars, Figure 2A).

The results summarized in Figure 2A therefore suggest the presence of one or more factors in the supernatant that restrict PM production. Furthermore, in the resuspended culture, PM production diminished with increasing OD from the point of harvest/resuspension until complete inhibition at OD >40. However, when samples taken at different OD levels were plated on minimal or lysogeny broth (LB) medium, all colonies had the PM-producing phenotype of the wild-type strain. Therefore, loss of PM production through mutation could be ruled out.

Another interesting observation was that fresh cells inoculated in culture supernatant grew with a higher initial growth rate than the control (aerobic cells/fresh cultivation medium, Figure 2B). However, this effect declined for cells cultivated in culture supernatants harvested at OD >25. These initial results showed that cells provided with fresh growth medium were capable of producing higher PM levels and that substances which accumulated in the culture supernatant have an influence on the initial growth rate and the PM production. As the changes in cell behaviour were strongly dependent on the culture density, we suspected that a quorum sensing system could be responsible for the observed phenomena. We hypothesized that both autoinducer signal molecules and bacteriochlorophyll *a* precursors might be present in the culture supernatant.

**Figure 3:**
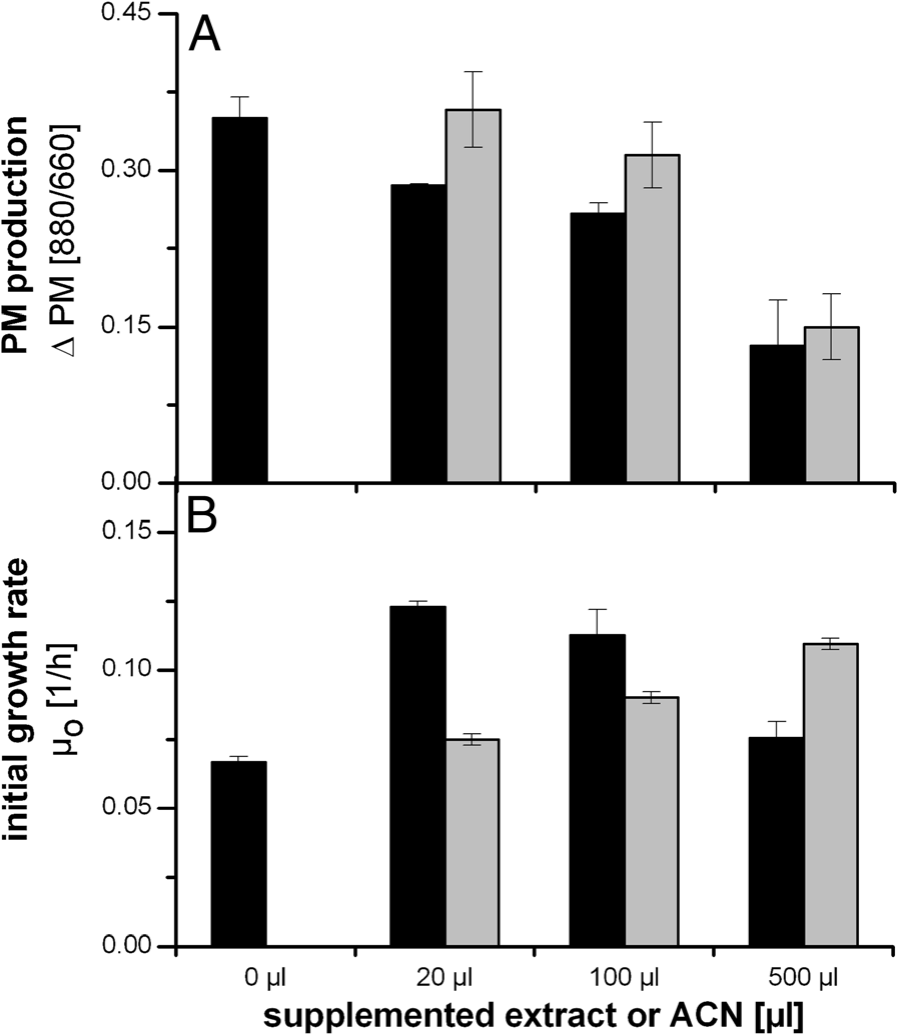
**Effect of different amounts of AHL extract on PM production (A) and initial growth rate (B) of *R. rubrum*.** Cell-free supernatants from the stationary phase of a microaerobic Fed-Batch cultivation, in which PM production is completely inhibited, were extracted with dichloromethane, evaporated to dryness and resuspended in acetonitrile (ACN). Different volumes of AHL extract (black bar) or ACN (gray bars) were added to the culture at the point of PM induction **(A)** or prior to inoculation **(B)**. Initial growth rates of cells were calculated from data obtained from the first 20 hours of the experiment. Growth conditions are comparable to those used for Figure 2. The shown data represent the average of two biological replicates (two shake-flask cultivations of each extract amount were cultivated at the same time. The extract used in this experiment was obtained from the harvest of one Fed-batch cultivation). Error bars were calculated by error propagation of the deviations of three equivalent experiments (for each experiment extracts from one Fed-Batch cultivation were supplemented to shake-flask cultures).

Therefore, we investigated the effects of Fed-Batch cultivation supernatant constituents, after extraction by dichloromethane, on growth and PM expression in *R. rubrum*. After removing the dichloromethane by evaporation, the dry residue was resuspended in acetonitrile (ACN). These extracts were then added to *R. rubrum* cultivations in flask experiments (Figure 3). The addition of extracts from *R. rubrum* cultures caused a strong reduction in PM production. To rule out that the effect was caused by the addition of ACN, pure ACN was added to control cultures. ACN alone slightly lowered PM synthesis if added in volumes larger than 20 μl. However, the ACN-containing culture extract produced significantly stronger effects. Addition of excess ACN (500 μL) diminished the effect of the extract.

In contrast to PM production, the initial growth rate (*μ*_0_) increased in proportion to an increasing volume of pure ACN (Figure 3B, grey bars). However, the ACN-containing *R. rubrum* extract stimulated the highest growth rate when added at 20 μL and the initial growth rate declined with an increasing extract volume. The addition of 500 μL extract appeared to retard the growth rate, although this effect was not observed with the same volume of ACN (Figure 3B).

We note that Figure 3B also shows a steadily increase in the initial growth rate of the control cultures when only increasing amounts of the solvent ACN were added. The growth stimulation strongly suggests that *R. rubrum* is capable of utilizing ACN as a source of carbon and/or nitrogen. A gene encoding a bifunctional nitrilase (YP_425830) is annotated in the genome sequence of the strain employed in our study.

After the addition of the 500 μL extract, the recipient culture contained comparable levels of extractable substances to those in the Fed-Batch culture at the time point of inhibition of PM production. (OD = 30 in Figure 1). Altogether, the results presented in Figure 3 underline the presence of at least one substance in the extract that restricts PM production, enhances growth at lower levels, and retards growth at higher levels.

To check if accumulated bacteriochlorophyll *a* precursors influence the PM synthesis by the cells, PPIX (chemically synthesized) and Mg-PPIX-mme (isolated from microaerobic HCD cultures supernatants) were added to a growing culture at OD = 1, the point at which PM synthesis is normally induced by oxygen depletion. Tetrapyrole precursors were supplemented in amounts equivalent to those observed under HCD conditions. Addition of either PPIX or Mg-PPIX-mme resulted in slightly lower PM levels compared to the control (MeOH) (see Additional file 1: Figure S1). However, the reduction was weaker than the effect caused by the addition of the culture extract or by resuspending fresh cells in culture supernatant.

### *R. rubrum* produces different types of bioactive AHLs

To check the *R. rubrum* cultures for bioactive AHL, sterile-filtered culture supernatant from a Fed-Batch HCD culture was analyzed with a thin layer chromatography bioassay with *Agrobacterium tumefaciens* NTL4 as an indicator strain [18]. These assays clearly demonstrated the bioactivity of *R. rubrum* HCD culture extracts with the TraR-dependent quorum sensing system of *A. tumefaciens* NTL4, indicated by intense blue spots on the agar-overlaid TLC plates (see Additional file 1: Figure S2).

**Figure 4:**
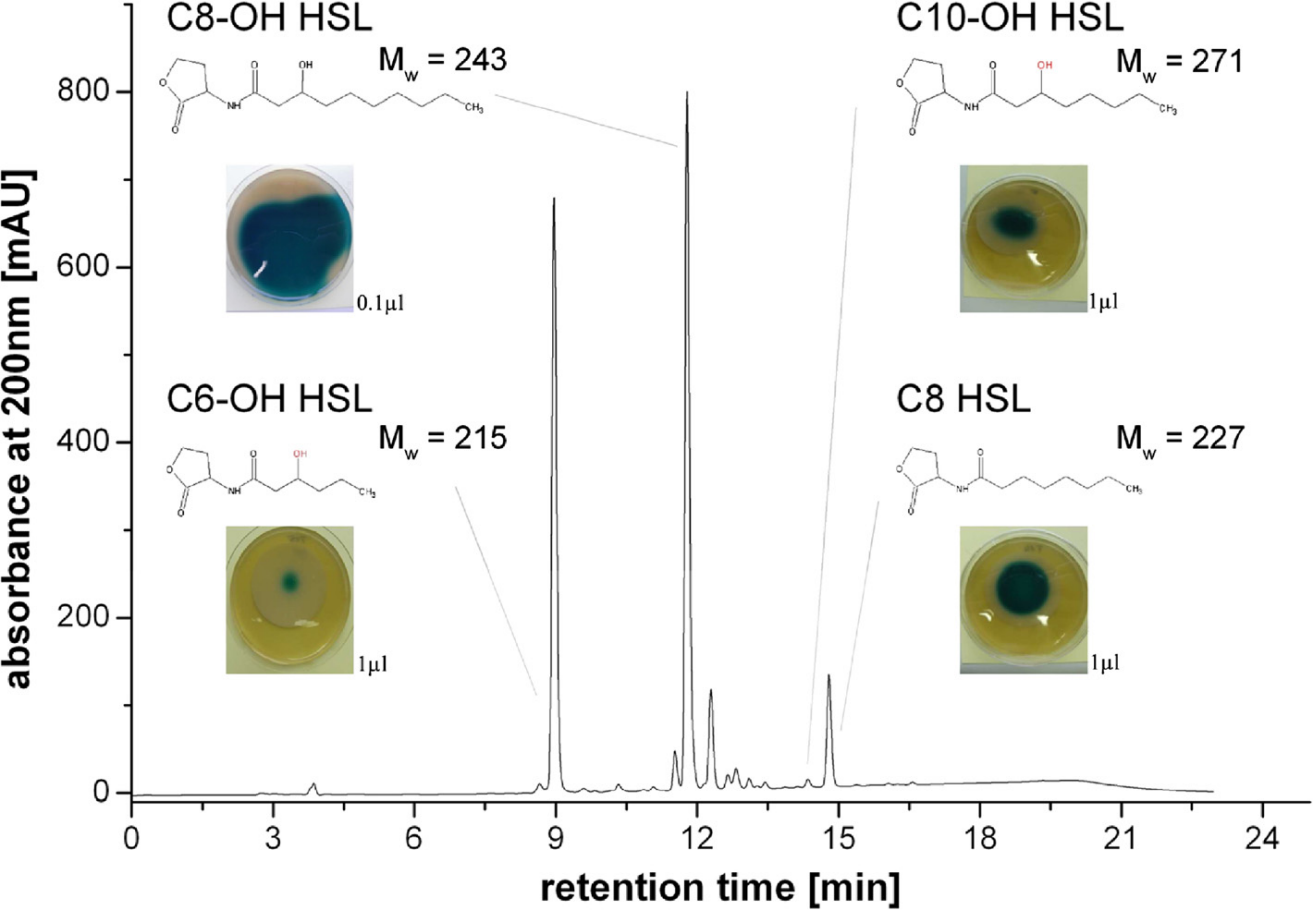
**HPLC separation of crude extracts obtained from *R. rubrum *Fed-batch culture supernatants at OD = 50.** Chemical structures and molecular weights (Mw) of identified AHLs are indicated (for a list of measured *m/z* values see supporting material). Single peaks were isolated by semi-preparative HPLC and applied to *A. tumefaciens* NTL4 on agar plates. The inserts show the biological activity as blue colour reaction. Volume of HPLC eluate loaded onto agar containing *A. tumefaciens* is indicated in μL.

The extracts were further examined by HPLC-MS for the presence of AHLs. For identification and quantification of HPLC peaks, a commercially available C8oxo-HSL and a derived C8OH-HSL (see Material and Methods) were employed as standards for comparison of retention time, MS signals and DAD spectral properties. In the reversed phase HPLC-separated extract, the following six AHLs could be identified in the supernatant of *R. rubrum* HCD cultures: *N-*(3-hydroxhexanoyl)-homoserine lactone (C6OH-HSL), *N-*(3-hydroxyoctanoyl)-homoserine lactone (C8OH-HSL), *N-*(3-octanoyl)-homoserine lactone (C8-HSL), *N-*(3-decanoyl)-homoserine lactone (C10-HSL), *N-*(3-hydroxydecanoyl)-homoserine lactone (C10OH-HSL) and *N-*(3-hydroxydodecanoyl)-homoserine lactone (C12OH-HSL) (for *m/z* values, see Additional file 1: Table S3). The concentration of C8OH-HSL in the supernatant of an aerobic Fed-Batch cultivation at OD = 50 was ~330 μM. The concentrations of the other AHLs were not determined due to the lack of a reference standard. Since only very small peaks of C10-HSL and C12OH-HSL were detected, these compounds were not considered further. The more abundant peaks were isolated by semi-preparative HPLC as pure fractions and applied to the *A. tumefaciens* NTL4 autoinducer bioassay on agar plates (Figure 4). C6OH-HSL, C8-HSL, C8OH-HSL, and C10OH-HSL caused a blue colour response of the indicator strain thus confirming the results obtained with crude dichloromethane extracts.

### AHL profiles at different growth modes

**Figure 5:**
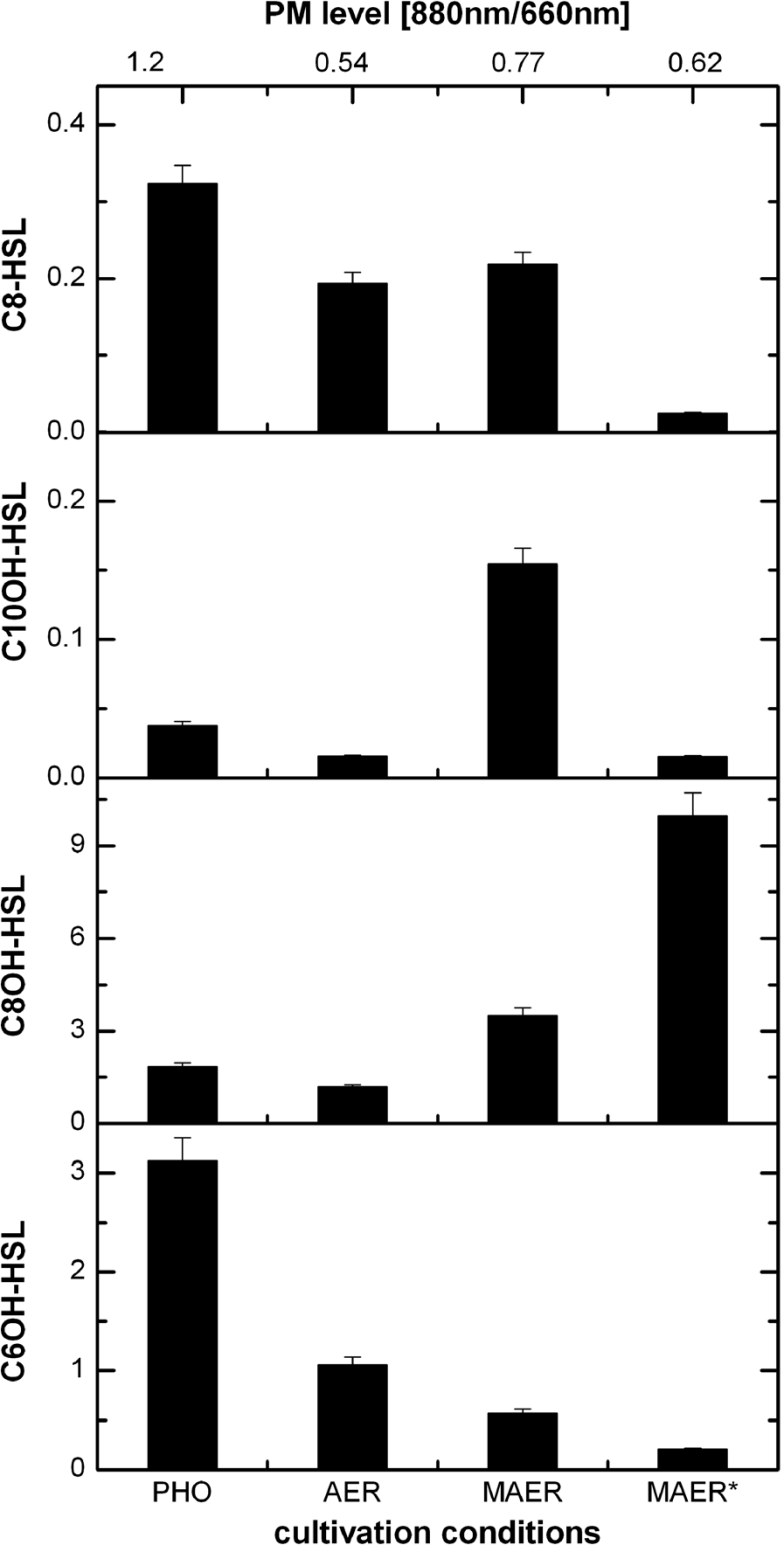
**AHL accumulation profiles of *R. rubrum *cultivated under different growth conditions.** AHL levels obtained from HPLC analysis are given in mAUsOD^-1^ ml^-1^ and are therefore qualitative estimates. AHLs were extracted from supernatants of cultures grown under phototrophic (PHO), aerobic (AE) and microaerobic (MAER) conditions. For microaerobic cultures, the supernatant was harvested at two time points. MAER* refers to a later harvesting point at which PM production has stagnated. Cultivations under aerobic and microaerobic conditions were performed in bioreactors, whereas phototrophic cultures were grown in pyrex bottles. At top of graph, values indicate PM levels at harvest. PM value of 1.2 represents maximum PM levels and a value of 0.54 indicates a complete lack of PM formation.

Since *R. rubrum* is a very versatile life-form capable of growing under anaerobic photosynthetic conditions as well as aerobically and microaerobically in the dark, we analyzed whether the different growth modes would be reflected in the AHL profiles (for details of growth conditions see Materials and Methods). Figure 5 presents relative AHL levels in the various cultures during exponential growth. To investigate if the inhibition of PM was correlated with the AHL profile, we extracted the AHLs at two points under microaerobic growth conditions: MAE indicates extraction during PM production and MAE* indicates extraction from an older MAE Fed-Batch culture when PM synthesis was already inhibited.

Strikingly, C8OH-HSL was the most abundant AHL in microaerobic cultures (Figure 5), and the sole AHL which was particularly abundant at later stages of the culture when PM production was already halted (MAE*). In phototrophic cultures with full PM expression, C8OH-HSL was the least abundant of all AHLs. In sharp contrast, C6OH-HSL was much higher in photosynthetic cultures than in microaerobic HCD cultures with repressed PM biosynthesis. C10OH-HSL was the only molecular species, elevated in PM-producing microaerobic (MAE) cultures. C8-HSL was present in all growth conditions in similar amounts except in microaerobic (MAE*) cultures where it was much lower. However, unlike the bioreactor cultivations in which the pH was stable, the pH in flask cultivations increased to ~8, which may alter stability of AHLs [23]. Accordingly, we observed differences in C6OH-HSL and C8OH-HSL accumulation between flask and bioreactor cultivations. Therefore, only data obtained from bioreactor cultivations are shown, in which pH was maintained at 6.8. Since these results suggested an important role for C8OH-HSL in microaerobic conditions, we investigated the phenotypic response of *R. rubrum* to this compound by adding purified C8OH-HSL to cultures grown microaerobically in M2SF medium. When applied at a concentration of 330 μM (corresponding to the concentration measured during Fed-Batch cultivations at the time point of PM inhibition) PM expression was significantly reduced to about 2/3 of the control culture. Reducing the applied concentration to 175 μM showed a weaker response in PM levels but slightly stimulated the growth rate of the culture. At 330 μM, no significant effect on growth was observed (see Additional file 1: Figure S3). These results highly support the assumption that the observed HCD effects are influenced by quorum sensing and that C8OH-HSL plays an important role in quorum sensing under microaerobic conditions.

### Identification of quorum sensing-related genes by genome sequence analysis

We performed a sequence homology based search in the genomic sequence of *R. rubrum*[24] by reference to known quorum sensing genes such as *luxR* and *luxI* from *V. fischeri*[25]. Results of the pBLAST algorithm indicate that *R. rubrum* possesses one LuxI homologue (YP_428477.1) and 6 LuxR homologues (YP_428476.1, YP_427022.1, YP_427266.1, YP_428311.1, YP_427687.1, YP_427319.1). Similar to many *luxRI*-type genomic arrangements, the *luxI* gene (Rru_A3396:3913528…3914148) is located in close proximity (154 bp downstream) to *luxR1* (Rru_A3395:3912592..3913374).

A pBLAST search for enzymes capable of degrading quorum sensing signal molecules found three proteins (YP_428352.1, YP_426609.1, YP_425120.1) with high homology to the lactonase AiiA [26] and one protein (YP_426927.1) with high homology to the acylase PvdQ [27] (see Additional file 1: Table S2).

### mRNA profiles of the lux-type quorum sensing system of *R. rubrum*

**Figure 6:**
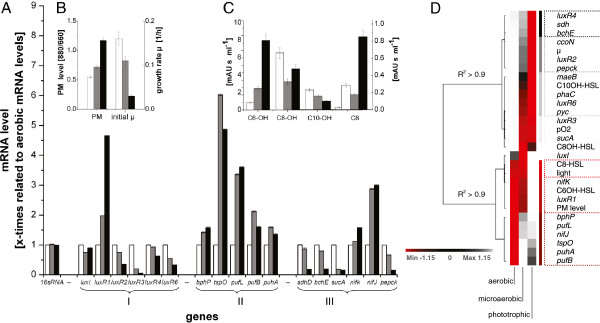
**Relationship between growth conditions and gene expression profiles of the *lux*-type genes in *R. rubrum. *A**: mRNA accumulation from selected genes in *R. rubrum* cultures grown under aerobic (white), microaerobic (grey) and phototrophic (black) conditions. **B**: PM levels and initial growth rates. **C**: AHL accumulation. All samples were harvested during exponential growth at an optical density of ~2. Genes associated with quorum sensing (I), the PM production (II), and metabolism (III) are indicated. **D**: Cluster analysis of growth condition dependent data shown in **A**, **B** and **C**. The red/gray pattern indicates the degree of structural identity; components with a high structural identity (R^2^ > 0.98) are clustered as indicated by the coloured groups (· · ··). Cluster analysis was performed using PermutMatrix version 1.9.3.

To investigate if the genes of the quorum sensing system are active and if a relationship between the accumulation of mRNA and AHL exists, mRNA levels of selected genes of *R. rubrum* cultures cultivated under aerobic, microaerobic and phototrophic conditions were analyzed by RT-PCR. Figure 6A shows the mRNA accumulation levels of the *lux* similar genes (I) and other genes which are either involved in PM production (II) or are key enzymes of the central metabolism (III). (The mRNA levels of *luxR5* are not included since all primer pairs for this gene showed unspecific PCR products.) The data presented in Figure 6 were obtained at low cell densities (OD ~2) and illustrate that the cellular mRNA levels of the respective *lux* genes differed in accordance to the growth conditions.

Correlation analysis of these measurements revealed significant cluster patterns (Figure 6D). At the beginning only clusters with a structural identity >0.98 were taken into account (refer to coloured groups in Figure 6D). *luxR1* expression was strongly correlated (R^2^ = 1) with both PM and C6OH-HSL levels and also with the expression level of *nifK* (Rru_A1012). Both *nifK* expression and PM production are strongly repressed in response to oxygen in *R. rubrum*[4,28]. The *luxR2* mRNA accumulation correlated with the initial growth rate (μ) and expression of the genes coding for phosphoenolpyruvate carboxykinase (*pepck)* and cytochrom oxidase cbb3 (*ccoN*). *luxR3* expression correlates with the oxygen availability (pO_2_) and the expression of alpha-ketoglutarate dehydrogenase. *luxR4* expression clustered with the expression of *bchE* and *sdhD* encoding Magnesium-Protoporphyrin IX monomethylesther (Mg-PPIX-mme) cyclase, an enzyme in the bacteriochlorophyll pathway, and the subunit D of the succinate dehydrogenase complex, respectively. *luxR6* clustered with C10OH-HSL and genes coding for poly(R)-hydroxyalkanoic acid synthase (*phaC)*, malic enzyme (*maeB*) and pyruvate carboxylase *(pyc).* These enzymes are involved in coordinating the metabolic fluxes of the central carbon metabolism relative to the available carbon source*.* C8-HSL clustered only with the availability of light. *luxI* and C8OH-HSL showed no significant correlation. If the coefficient describing the structural identity in Figure 6D is relaxed to a value of 0.9, the data falls into two groups. The lower group contains *luxR1* and C8-HSL along with *bphP, tspO, pufL puhA* and *pufB* which are known to be related to PM formation in other anoxgenic photosynthetic bacteria*.* In contrast, the upper group contains both the remaining *luxR*-similar genes and genes encoding enzymes which are involved in growth modes and regulation of related metabolism.

### Dynamics of the quorum sensing system during Fed-Batch cultivation

**Figure 7:**
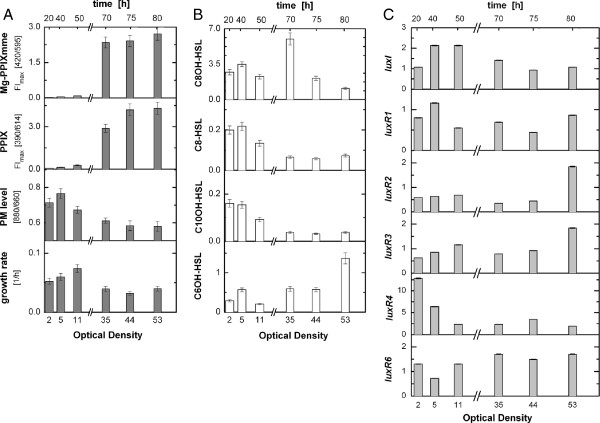
**Dynamics of a microaerobic HCD cultivation of *R. rubrum*.** Measurements were made at multiple time points of a growing culture (indicated by increasing optical density). **A**: growth rate, PM production, protoporphyrin IX (PPIX) and Mg-protoporphyrin IX monomethylester (Mg-PPIXmme) accumulation. **B**: Relative amounts of accumulated AHL in mAUsOD-1 ml-1. **C**: Accumulation of mRNA from selected *lux*-type genes. mRNA levels are related to the expression of these genes in aerobically grown *R .rubrum* cultures at an OD of 2. These data was obtained from the Fed-Batch cultivation shown in Figure 1.

For a comprehensive picture of the contribution of the quorum sensing system to HCD cultivations of *R. rubrum*, the expression of *lux* genes and the kinetics of AHLs were monitored throughout the time course of a microaerobic Fed-Batch cultivation and correlated to PM expression and growth rate (Figure 7). The accumulation of the tetrapyrolle compounds PPIX and Mg-PP-mme in the culture broth was also determined. At population densities, OD ≤ 11, accumulation of *luxR1* and *luxI* mRNAs correlate with the appearance of C8-HSL, C10OH-HSL and C8OH-HSL and PM levels (Figure 7). Accordingly, with an increasing cell density, the PM production and the accumulation of C8-HSL, C10OH-HSL, *luxR1* and *luxI* mRNA decreased. The time-point of rapid and substantial C8OH-HSL accumulation coincided with the accumulation of both PPIX and Mg-PPIX-mme, a significant decrease in the growth rate and PM inhibition. During the following period, at the highest population density, the most abundant AHL was C6OH-HSL accompanied by elevated levels of *luxR2* and *luxR3* transcripts. The mRNA of *luxR6* showed no significant variation during the entire cultivation.

## Discussion

### PM production and growth rates appear to be regulated by quorum sensing

HCD cultivations of *R. rubrum* are an important precondition for the industrial production of photosynthetic compounds, as this organism is capable of expressing maximum levels of PM independent of light in large scale bioreactors. The application is, however, severely hampered by the apparent loss of *R. rubrum*s capacity to produce PM under HCD conditions [11].

In the present study we demonstrate that the PM inhibition in HCD cultures can be attributed to the accumulation of soluble factors, accumulating in the culture supernatants during cultivations of *R. rubrum.* We suggest that the attenuation of the PM synthesis is quorum-related, as the inhibition of PM biosynthesis increased with an increasing OD level. Moreover, we observed the quorum-dependent attenuation of the PM synthesis also for cells which were washed and resuspended in fresh medium. Since we excluded cell mutation as potential reason, we assume that the composition of the culture broth aliquot is reconstructed, after cells are transferred, in a manner that is dependent on cell density. The supplementation of organic solvent extracts from HCD cultures to *R. rubrum* supports these findings as the inhibition of PM was stronger when higher amounts of extract were supplied. Depending upon the supplied extract amount, growth rates either increased or decreased in response to the supplementation.

Several lines of evidence suggest that the metabolites responsible for these effects are quorum related. Firstly, culture supernatant extracts were shown to contain high levels of AHLs. The most abundant of these was C8OH-HSL. Also present were C6OH-HSL, C8-HSL and C10OH-HSL, and trace levels of C10-HSL and C12OH-HSL. Biological activity was demonstrated using an *Agrobacterium tumefaciens* indicator strain. Secondly, when added to *R. rubrum* cultures, their effect was to reproduce and strengthen the responses of PM production and growth rates.

In the related species *Rhodobacter sphaeroides*, a single AHL (7,8-cis-N-(tetradecenoyl)-HSL) has been reported so far, apparently associated with polysaccharide formation and cell aggregation [12]. However, to our knowledge, the present study is the first report showing that AHL autoinducer molecules correlate with photosynthetic gene expression in anoxygenic photosynthetic bacteria and the first profiling of AHLs at different growth modes in these bacteria. In particular, the extreme heterogeneity in the abundance of the individual molecular species in phototrophic vs. chemotrophic grown cells suggest that these compounds contribute to the versatile physiological adaptation of this organism to changing light and oxygen conditions. In particular, the appearance of C8OH-HSL at later stages of Fed-Batch cultivations and general correlation with PM repression in microaerobic cultures, in combination with the respective effect when the purified compound is applied to *R. rubrum*, makes C8OH-HSL a major candidate as a signaling molecule involved in PM formation under microaerobic conditions. We cannot exclude at present that the six AHLs identified in this study do not reflect the complete repertoire of AHLs synthesized by *R. rubrum*. The employed HPLC elution profile might have missed for example low chain length (C4-HSLs) and/or long chain (C14-HSL) compounds as well as AHLs of very low abundance. Based on our results, C6OH-HSL during phototropic growth with fully expressed PM, and C8OH-HLS in microaerobic chemotrophic cells with inhibited PM expression appear to be major complementary players in the contribution of quorum sensing to photosynthetic gene expression.

Moreover the results of the present study suggest that AHL levels can significantly influence growth rates. It has been reported that bacteria with acyl-HSL-degrading activity can grow on a basal growth medium containing 3-oxo-hexanoyl-L-HSL as the sole carbon and nitrogen source [29,30]. As *R. rubrum* possesses homologues of AHL degrading proteins (PvdQ and AiiA homologues, see Additional file 1: Table S2), we expected the enhanced growth to be related to an additional supply of carbon source. However, as higher AHL amounts seem to suppress the initial cell growth the observations of Chan et *al.*[30] and Leadbretter et *al.*[29] seems to be inadequate to explain the observed behavior. Therefore, these results suggest a non-nutritional role for AHLs in their effect on growth rates.

### Effect of bacteriochlorophyll a precursor on PM synthesis

During the previous development of HCD Fed-Batch cultivation for *R. rubrum*, we noticed that tetrapyrrole precursors of the bacteriochlorophyll a biosynthetic pathway accumulated in the cultivation broth [11]. It is therefore possible that these compounds have an inhibitory effect on PM expression in addition of alternatively to AHLs. In the present study, under microaerobic HCD conditions, PPIX and Mg-PPIX-mme accumulated in the culture supernatant when PM synthesis is completely inhibited (Figure 7A). In contrast, under aerobic HCD conditions, Mg-PPIX-mme was the only precursor molecule which was detected in the culture supernatants [11]. Interestingly, in our experiments the accumulation of all the tetrapyrrole pigments coincided with the use of pure oxygen as input variable to control the oxygen-tension (data not shown). In this context, Yeliseev *et al.* proposed that the tetrapyrrole pigments accumulate in the culture supernatant of *R. sphaeroides* in response to the availability of molecular oxygen and that these pigments are capable of repressing the expression of genes encoding enzymes and structural polypeptides required for the PM synthesis in a modest but consistent manner [31,32]. In experiments on *R. rubrum*, we also observed a weak effect on PM production upon supplementing microaerobic flask cultures with Mg-PPIX-MME and PPIX (see Additional file 1: Figure S1). However, PM production was not completely suppressed, as is the case in HCD cultivations. Therefore we conclude that the accumulation of these pigments may provide a minor contribution to the repression of PM synthesis but is unlikely to be the major initiator. Rather, most of the suppression of PM production at OD >40 is caused by a combination of both AHLs and tetrapyrrole pigments. Alternatively, pigment accumulation may itself be regulated by quorum sensing.

### *R. rubrum* is equipped to sense its quorum

A pBlast analysis identified genes in *R. rubrum* which are highly homologous to known components of quorum sensing in other bacteria. Based on this approach, *R. rubrum* has one LuxI type AHL synthase, six LuxR-type regulators, three AiiA lactonases and one PvdQ lactonase. We detected significant amounts of mRNA of the *luxI* homologue and of five *luxR*-type homologues which demonstrates that these genes are expressed in *R. rubrum* (see Figure 6)*.* Further gene expression analysis suggested that the quorum sensing system in *R. rubrum* might be involved in the adaptation of the metabolism under distinct growth modes.

For the more detailed exploration of the apparent complexity of quorum sensing system in *R. rubrum* and validation of the conclusions of the present phenomenological study continuing work will be necessary. These next steps will include a set of knock-out mutants where individual components of the quorum-sensing circuit have been deleted and their phenotypic characterisation.

### An ecological point of view

From an ecological point of view, quorum sensing-dependent behavior is expected to play a role in the survival of bacteria. Thus, the observation that AHLs in *R. rubrum* cultures affect growth rate and PM expression suggests a survival strategy of this bacterium in its aqueous habitat. If growth is allowed to continue unchecked, the inevitable deficiency of resources may lead to an overall reduction in fitness of a population [33]. At high cell densities light becomes a limiting factor and it might be favourable to reduce the light harvesting capacity when cellular energy can be generated by microaerobic oxidative phosphorylation. Therefore, the light harvesting capacity of the PM would be expected to be reduced in high density populations, hence the restriction in PM production by AHL accumulation. Unlike other anoxygenic photosynthetic bacteria, *R. rubrum* seems to lack a light sensing system and therefore may rely quorum sensing for this control. It is long known that limting oxygen is the primary environmental factor for inducing photosynthetic gene expression, However, under anaerobic conditions, the expression of PM shows an inhibition by high light intensities while maximal amounts are produced at low light intensities. The molecular basis for the light-regulation is not well understood as no specific light-sensor was found so far in *R. rubrum*.

## Conclusions

In this work, we analyzed the growth behavior of *R. rubrum* cultures, during microaerobic Fed-Batch cultivations, to investigate the cause of the recently observed HCD effects. Our results show that these effects are quorum-related and that they can be correlated to the accumulation of high amounts of bioactive AHLs in the culture supernatant. Clearly, these findings are to be taken into account whenever the industrial production of compounds associated with PM formation under HCD conditions of *R. rubrum* is considered.

## Abbreviations

AER: Aerobic; AHL: *N*-acyl-homoserine lactone; CRP: Culture redox potential; HCD: High cell density; HSL: Homoserine lactone; MAE: Microaerobic; Mg-PPIX-mme: Mg-protoporphyrine-IX-monomethylester; OD: Optical density; PHO: Phototrophic; PPIX: Protoporphyrine-IX; PM: Photosynthetic membranes; pO2: Partial oxygen pressure; TLC: Thin layer chromatography.

## Competing interests

The authors declare that they have no competing interests.

## Authors’ contributions

LC conducted the laboratory work on *R. rubrum* cultivations, gene expression analysis and bioindicator assays, sample preparation for HPLC analysis, collated and analyzed the data; AC participated in running the experiments and conducted the AHL analytic; LC and AC conceived of the study; MM and HG participated in its design and coordination. LC and MM drafted the manuscript. All authors contributed to, read, criticize and approve the final manuscript.

## Supplementary Material

Additional file 1 Supplemental Material. Click here for file
