# Supplementary material for: Quorum sensing influences growth and photosynthetic membrane production in high-cell-density cultivations of Rhodospirillum rubrum
Source: BMC Microbiol. 2013 Aug 8;13:189. doi: 10.1186/1471-2180-13-189 (PMC3751510; doi:10.1186/1471-2180-13-189)
Supplement: Additional file 1 — Supplemental Material. [file 1471-2180-13-189-S1.docx]

Quorum Sensing influences growth and photosynthetic membrane production in High-Cell-Density Cultivations of *Rhodospirillum rubrum*

L. Carius, A. B. Carius, M. McIntosh and H. Grammel

**Supplemental Material:**

Supplemental Table 1: List of primer sequences. Index *: Homologue of the multi-sensor signal transduction histidine kinase BphP of *P. aeruginosa* with an e-value of 4e-53.

Supplemental Table 2: Phylogenetic comparisons of *R. rubrum* quorum sensing related genes and other relevant aspects of the *R. rubrum* lifestyle*.* Sequences were aligned by CLUSTALW. Only hits with a query coverage > 90 % and an E score < 3.00E-15 are shown. (see additional data file )


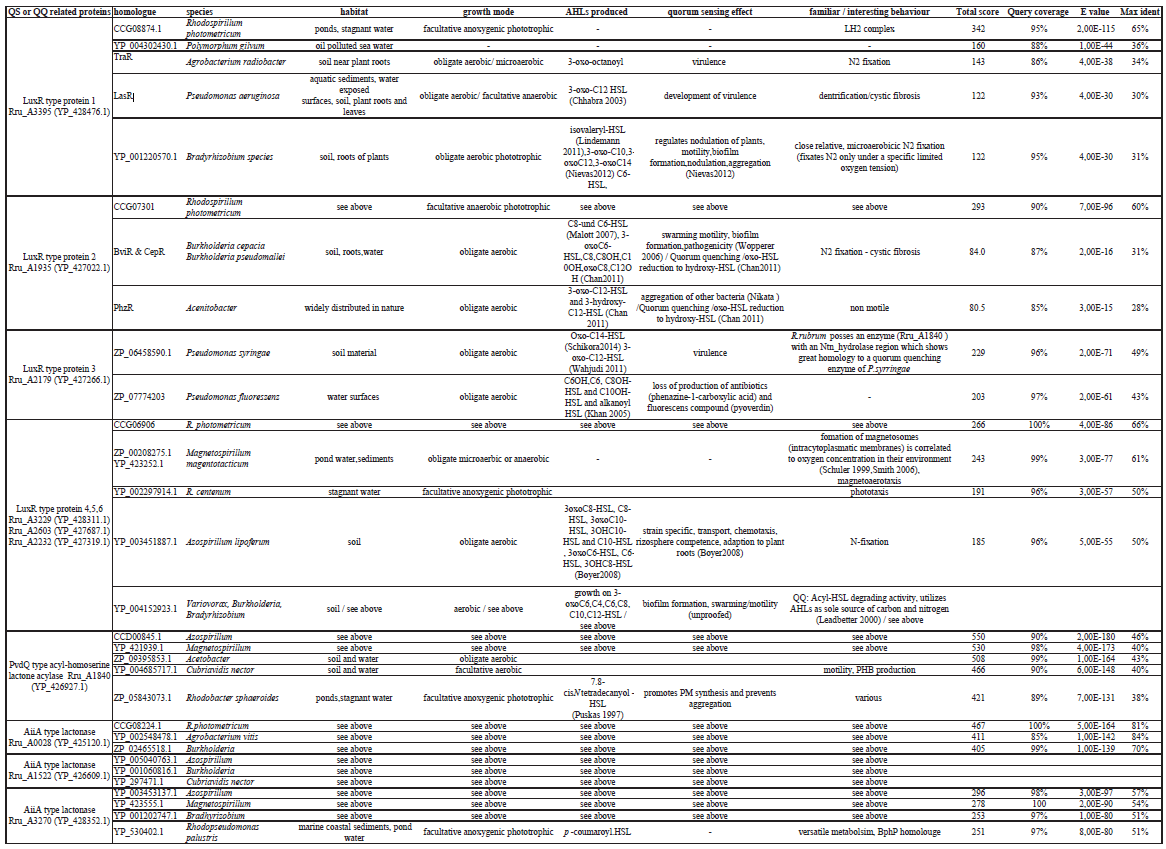


Supplemental Table 3: Table of AHLs detected during the study. In all cases, the masses were detected in positive as well as in the negative scan. The use of acetonitrile and desalted milli-Q-water in combination with an APCI ion source lead to mostly protonated and deprotonated ions. Na-adducts were not commonly detected.

| **Substance** | **Short name** | **Retention time [min]** | **MW** | ***m/z*** | | **Detected Mass** | |
| --- | --- | --- | --- | --- | --- | --- | --- |
|  |  |  |  | **protonated** | **deprotonated** | **protonated** | **deprotonated** |
| *N-*(3-hydroxhexanoyl)-homoserine lactone | C6OH-HSL | 5,8 | 215 | 216 | 214 | 216.0 | 214.4 |
| *N-*(3-hydroxyoctanoyl)-homoserine lactone | C8OH-HSL | 8,9 | 243 | 244 | 242 | 244.0  245.2 * | 242.4  243.2 * |
|  |  |  |  |  |  |  |  |
| *N-*(3-octanoyl)-homoserine lactone | C8-HSL | 11,9 | 227 | 228 | 226 | 228.0 | 226.4 |
| *N-*(3-hydroxydecanoyl)-homoserine lactone | C10OH-HSL | 11,5 | 271 | 272 | 270 | 272.0 | NA |
| *N*-(3-decanoyl)-homoserine lactone | C10-HSL | 15,2 | 255 | 256 | 254 | 256.0 | NA |

* Isotopes that contain one heavy C13 isotope for C8OH-HSL (MW: 243), cf. Supplemental Figure 4.


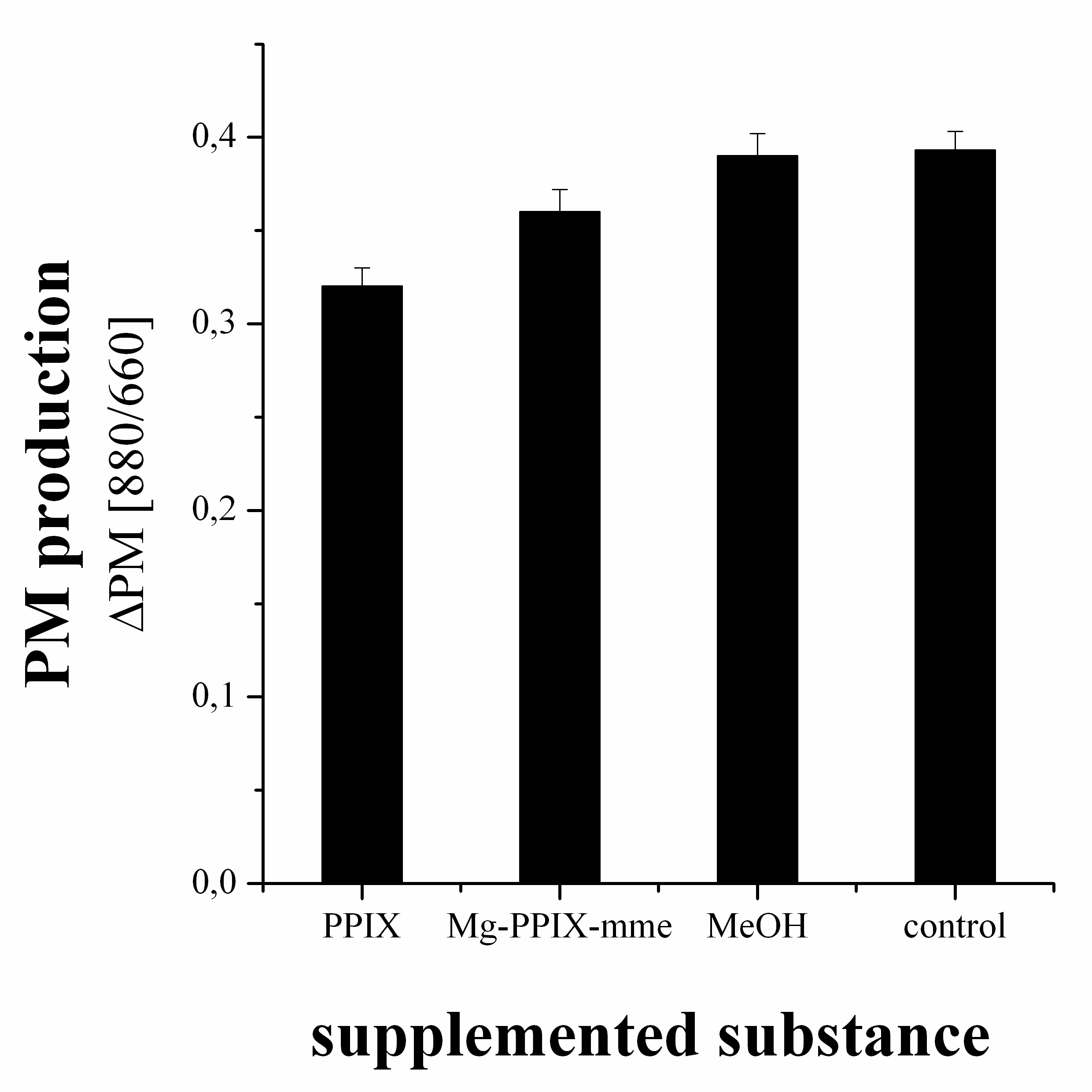


Supplemental Figure 1: Effect of tetrapyrrol pigments on PM formation in *R. rubrum* during microaerobic growth. Synthesized PPIX, Mg-PPIX-MME isolated from aerobic HCD cultures or pure MeOH as control were added to the culture at time point of PM synthesis induction at an OD of 1. Growth conditions are comparable to those of Figure 2.


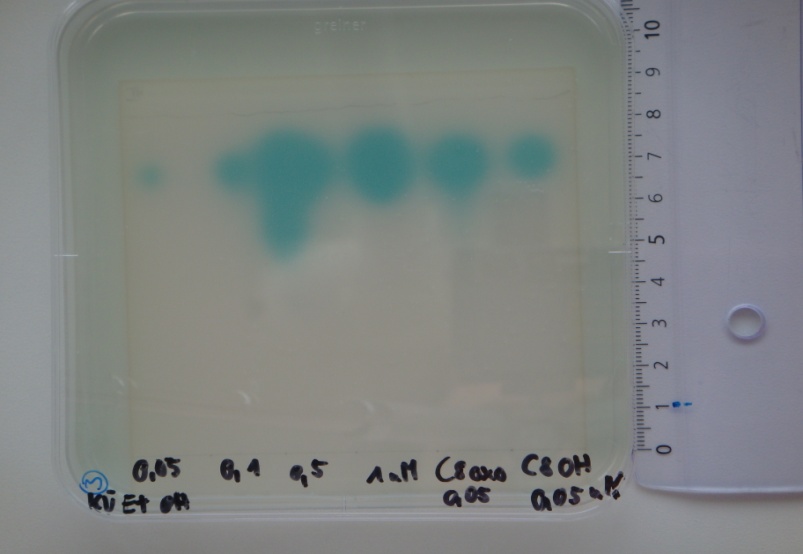


**nM: 0,05 0,1 0,5 1 5 0,05**

R1

R2

Supplemental Figure 2: Biosensor assay: Solvent dependent separation of AHLs with different chain length via Biosensor - Thin Layer Chromatography (TLC) (Luo 2001). Dichloromethane extracted AHLs resuspended in Ethanol were separated by TLC, overlaid with agar containing *Agrobacterium tumefaciens* NTL4. References R indicate commercial AHLs of different functionality. (R1 =C8-oxo, R2=C8-OH).


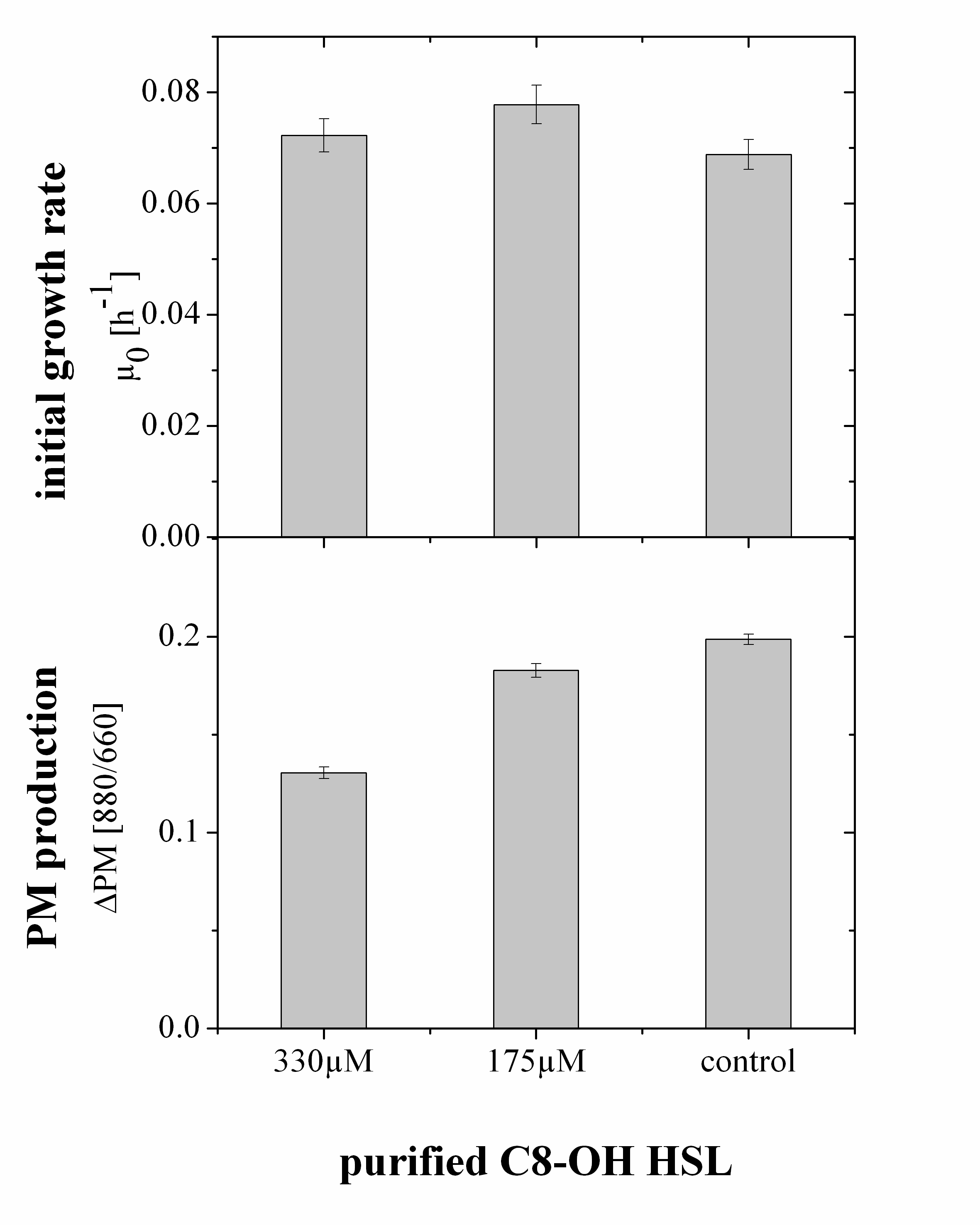


Supplemental Figure 3: Effect of purified C8-OH – HSL on cell growth (A) and PM formation (B) in *R. rubrum* during microaerobic conditions. Two amounts of synthesized C8-OH – HSL dissolved in ACN or pure ACN as control were added to the culture at time point of PM synthesis induction at an OD of 1. Error bars represent the relative standard deviation of the measurement method. For the sake of cost reduction the experiment was performed in test tubes to obtain smaller culture volumes. These change altered the oxygenation of the culture compared to Figure 2,3,4. Beside this growth conditions are comparable to those of Figure 2.

A

A

B

Supplemental Figure 4: Example for mass detection for AHL identification in extracts from *R. rubrum*. Masses for the protonated (A) and deprotonated (B) forms of the molecule were detected in their respective channels. Isotopes that contain one heavy C13 isotope for C8OH-HSL (MW: 243) are shown as well. At the respective retention time of 8.9 min, these are the most prominent molecular mass detected.
